# Successful treatment of HIV-associated multicentric Castleman's disease and multiple organ failure with rituximab and supportive care: a case report

**DOI:** 10.1186/1752-1947-4-32

**Published:** 2010-01-30

**Authors:** Robin H Johns, Tomas Doyle, Marc C Lipman, Kate Cwynarski, Joanne R Cleverley, Peter G Isaacson, Steve Shaw, Banwari Agarwal

**Affiliations:** 1 Department of Intensive Care, Royal Free Hospital, London, UK; 2 Department of Thoracic and HIV Medicine, Royal Free Hospital, London, UK; 3 Department of Haematology, Royal Free Hospital, London, UK; 4 Department of Clinical Radiology, Royal Free Hospital, London, UK; 5 Departments of Histopathology and Cytopathology, Royal Free and University College London Medical School, London, UK; 6 Departments of Anaesthesia and Intensive Care, Royal Free Hospital, London, UK

## Abstract

**Introduction:**

Multicentric Castleman's Disease (MCD), a lymphoproliferative disorder associated with Human Herpes Virus-8 (HHV-8) infection, is increasing in incidence amongst HIV patients. This condition is associated with lymphadenopathy, polyclonal gammopathy, hepato-splenomegaly and systemic symptoms. A number of small studies have demonstrated the efficacy of the anti-CD20 monoclonal antibody, rituximab, in treating this condition.

**Case presentation:**

We report the case of a 46 year old Zambian woman who presented with pyrexia, diarrhoea and vomiting, confusion, lymphadenopathy, and renal failure. She rapidly developed multiple organ failure following the initiation of treatment of MCD with rituximab. Following admission to intensive care (ICU), she received prompt multi-organ support. After 21 days on the ICU she returned to the haematology medical ward, and was discharged in remission from her disease after 149 days in hospital.

**Conclusion:**

Rituximab, the efficacy of which has thus far been examined predominantly in patients *outside *the ICU, in conjunction with extensive organ support was effective treatment for MCD with associated multiple organ failure. There is, to our knowledge, only one other published report of its successful use in an ICU setting, where it was combined with cyclophosphamide, adriamycin and prednisolone. Reports such as ours support the notion that critically unwell patients with HIV and haematological disease *can *benefit from intensive care.

## Introduction

The spectrum of HIV-associated disease on the ICU has changed markedly since the widespread adoption of combination antiretroviral therapy (Highly Active Antiretroviral Therapy, HAART) in the late 1990s. Whilst the incidence of opportunistic infections has decreased, that of several neoplasms, including Multi-centric Castleman's Disease (MCD) and Hodgkin's lymphoma is increasing. MCD is a lympho-proliferative disorder associated with Human Herpes Virus-8 (HHV-8) infection, characterised by fever, lethargy, anaemia and lymphadenopathy. Lymph node histology typically reveals angiofollicular hyperplasia and plasma cell infiltration. There is as yet no accepted therapeutic gold standard for MCD. Initial treatment approaches involved chemotherapy with agents such as vinblastine, etoposide and doxorubicin plus corticosteroids. More recently the anti-CD20 monoclonal antibody, rituximab, has been used. This targets HHV-8-infected plasmablasts, which co-express the B cell antigen CD20. Small case series of patients with MCD have shown rituximab to be an effective therapy in patients that do not require organ support on the intensive care unit [1-3].

## Case Presentation

A 46 year old Zambian woman was referred from another hospital with a 4 week history of fevers, night sweats, vomiting, diarrhoea, and renal impairment. She had been diagnosed HIV positive in 2005, and started on HAART one year later. She had previously been treated for *Herpes simplex *virus infection, *Cytomegalovirus *pneumonitis, and *Pneumocystis jirovecii *pneumonia (PCP). At referral her blood CD4 count was 480 × 10^6^/L (range in HIV negative populations, 400-1500 × 10^6^/L); and she had an undetectable plasma HIV load. On arrival at our centre, she was confused, and had obvious pitting oedema of both lower limbs, widespread lymphadenopathy, and hepato-splenomegaly. Investigations (Table 1) revealed anaemia, leucocytosis, thrombocytopaenia, and acute renal and liver dysfunction.

**Table 1:** Laboratory investigations on admission to Royal Free Hospital Haematology unit

Haemoglobin 8.4 g/dl	Urea 56 mmol/l
White Cell Count 17 × 10^9^/l	Creatinine 367 μmol/l
Neutrophils 13 × 10^9^/l	Bilirubin 101 μmol/l
Platelets 43 × 10^9^/l	Aspartate transaminase 185 IU/l
Prothrombin time 20.5 seconds	Albumin 18 g/l
Fibrinogen 5.4 g/l	Lactate 8.6 mmol/l
C-reactive protein 140 mg/l	

**Figure 1:**
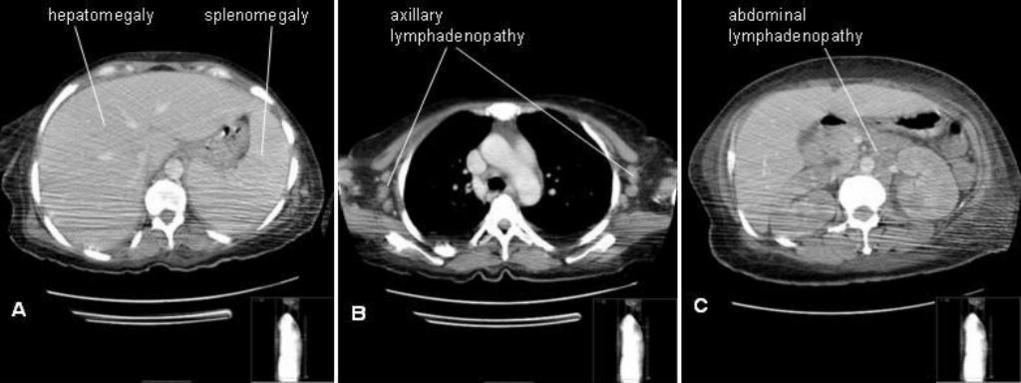
**Multi-detector computed tomography (CT) thorax and abdomen with intravenous contrast enhancement showing hepatosplenomegaly, axillary and abdominal lymphadenopathy**.

**Figure 2:**
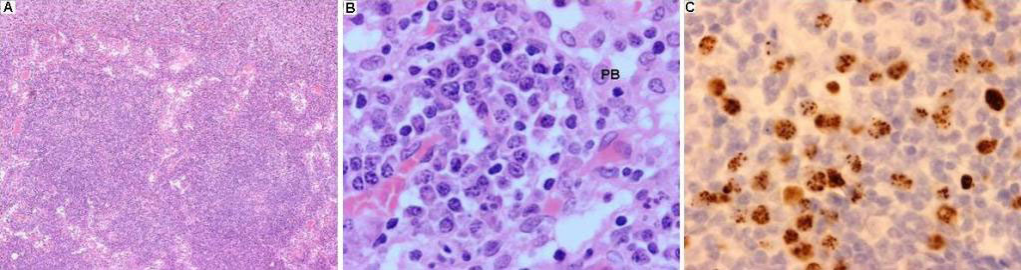
**Inguinal lymph node biopsy: histology characteristic of MCD**: **(A) **Haematoxylin & Eosin stained section of lymph node (original magnification × 2.5) showing effaced architecture with few residual follicles. **(B) **infiltration with large blastic cells (PB, original magnification × 60). **(C) **Immunostaining reveals blastic cells express HHV-8 (positive cells are brown, original magnification × 60).

A CT scan showed hepato-splenomegaly and gross lymphadenopathy involving the thorax, abdomen and pelvis (Figure 1). Inguinal lymph node excision biopsy confirmed the clinical suspicion of Multi-centric Castleman's disease (MCD) (Figure 2). Rituximab (375 mg/m^2^) together with hydrocortisone and rasburicase, was administered as specific treatment. She developed rapidly progressive metabolic acidosis, oliguria, and rising serum creatinine and was admitted to the ICU for haemofiltration. Antiretroviral therapy was continued on the ICU with ritonavir-boosted lopinavir and saquinavir. Abacavir and lamivudine, which the patient was already taking, were stopped because of their association with lactic acidosis and hepatic steatosis.

Following admission to ICU she rapidly became hypotensive, hypoglycaemic, coagulopathic and more anaemic. A possible basis for this could have been the systemic manifestations of a "cytokine storm" associated with MCD; increased expression of IL-6 is typical of MCD. Vasopressor and inotropic support with noradrenaline and dobutamine was required to maintain an adequate mean arterial pressure (MAP). Because of rapidly escalating requirements for noradrenaline she received a continuous infusion of hydrocortisone (10 mg/h) as per local departmental protocol, to treat probable relative adrenal insufficiency. Empirical antibiotics and antifungal agents were given to treat sepsis as a potential cause for ensuing multi-organ dysfunction, and she required a continuous infusion of 20% dextrose for refractory hypoglycaemia. To treat her acute renal failure and profound metabolic acidosis (serum lactate of 18.5 mmol/L), haemofiltration was undertaken with large volume 5 litre cycles (~90 ml/kg/hour) of lactate-free replacement fluid. This strategy was adopted to target early shock reversal and removal of IL-6, increased expression of which is a hallmark feature of MCD.

**Figure 3:**
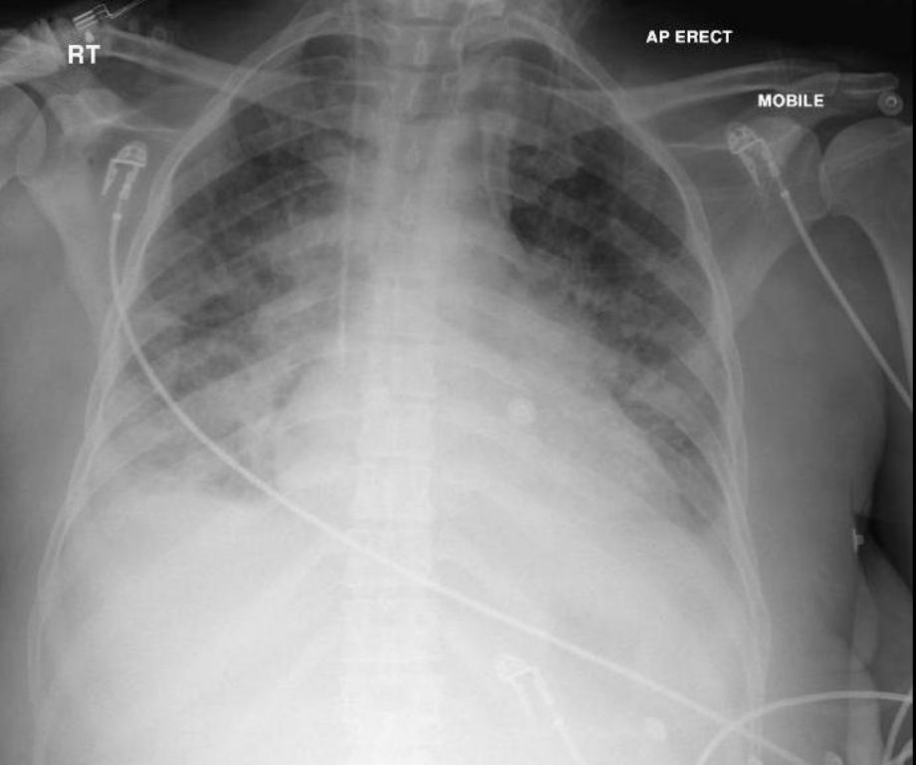
**Chest X-ray showing bilateral diffuse infiltrates**.

The chest radiograph progressed over four days to bilateral diffuse patchy consolidation (Figure 3), associated with greatly increased oxygen requirements (FiO2 0.8), and consistent with a diagnosis of acute respiratory distress syndrome (ARDS). The patient became drowsy and hypercapnic. Her trachea was therefore intubated, and mechanical ventilation was commenced.

She developed epistaxis and bleeding from insertion sites of arterial, central venous, and haemofiltration catheters. She had a positive direct Coombs' test consistent with autoimmune haemolytic anaemia (AIHA), a recognised association of MCD. In addition she had elevated prothrombin (PT) and activated partial thromboplastin times (APTT), reduced platelets and reduced serum fibrinogen consistent with disseminated intravascular coagulation (DIC). She received methylprednisolone, folinic acid, and red cell concentrate to treat anaemia; plus cryoprecipitate, fresh frozen plasma, vitamin K, and platelets for DIC. In addition to this extensive physiological support, her Castleman's disease was treated with weekly infusions of the anti-CD20 monoclonal antibody, rituximab, for four weeks.

From day 10 there was evidence of clinical improvement. She had a tracheostomy in the second week of her ICU stay, and she was slowly weaned from inotropic/vasopressor, ventilatory, and finally renal support. At day 21 of her ICU admission, she was discharged to the ward to complete her treatment with rituximab, and to continue rehabilitation from global muscle weakness, and reduce dependence on her tracheostomy. The patient was discharged home, in remission from her disease, after 149 days in hospital. When last seen in clinic she remained in remission and living independently 14 months from her treatment.

## Discussion

Survival of patients with HIV admitted to the ICU has improved substantially in the last 10 years, with HIV patients now being able to expect a similar chance of survival through to hospital discharge as general medical patients admitted to the ICU. The basis for this improvement is likely to be multifactorial, reflecting better understanding of HIV-associated disease, the availability of new combination antiretroviral therapy, the improved care of HIV patients both outside and within the ICU, and protective ventilation strategies for HIV patients with respiratory failure and acute lung injury.

Prognostic factors previously shown to be associated with a poor outcome in HIV patients admitted to ICU are low CD4 count and advanced HIV disease stage, acute illness severity (SAPS I: simplified acute physiology score I; or APACHE II: acute physiology & chronic health evaluation II), and the need for, and duration of, mechanical ventilation whilst on the ICU [4]. In addition, a haematological malignancy also independently further confers a poor prognosis. Such patients typically have severely impaired host defences and the undertaking of invasive procedures, such as endotracheal intubation and central venous cannulation, carries a major risk of infection.

Cornet *et al *[5] reported an ICU mortality rate of 60% for haemato-oncological patients compared with a rate of 27% for general critically ill patients. They also highlighted the poor long-term prognosis, with a 1 year mortality of 88% for haemato-oncological patients. However, such patients may not fare so badly on ICU if organ support facilitates administration of a specific therapy for a treatable condition. Benoit *et al *[6] have recently described successful outcomes in severely ill patients with haematological malignancies who receive intravenous chemotherapy in intensive care. Hence admission to ICU for specific therapy *can *be lifesaving.

The gold standard therapy for HIV-associated MCD is yet to be established. Vinblastine, etoposide and doxorubicin plus corticosteroids have all been used previously. Etoposide has been shown to be effective with resolution of systemic symptoms during its administration. However it has not been associated with prolonged remission. Interruption of chemotherapy usually results in clinical recurrence and most patients remain chemotherapy-dependent for life. In addition its use can be associated with cytopaenias. The use of etoposide would have been considered as adjuvant therapy in our case had the MCD not responded to rituximab monotherapy. In contrast rituximab is well tolerated and has been shown to be effective in case reports and series [1-3]. The role of rituximab in therapy for critically ill patients is less well established. In previously published reports, those admitted to ICU did not survive. Recently, however, a successful outcome of rituximab, in conjunction with cyclophosphamide, adriamycin and prednisolone, in the ICU setting, has been described [7].

In our patient, the clinical presentation was consistent with a systemic illness with a large inflammatory/infective component. The patient was originally from a country with endemic mycobacterial and fungal infection. Investigations therefore were undertaken to exclude a variety of possible causes - including disseminated mycobacterial and fungal disease, as well as lymphoma-like conditions, such as MCD. Prompt diagnosis was made following lymph node biopsy. The decision to biopsy an inguinal lymph mode, rather than a cervical node was based on the clinical findings of a large and easily palpable inguinal lymph node mass. Although small volume bilateral cervical lymphadenopathy was present, this was far less clearly abnormal than the groin lymph nodes. A particular issue in HIV patients is the presence of persistent generalised lymphadenopathy, which represents an immune response directed against HIV. This typically results in findings similar to the cervical adenopathy present in this patient; and therefore neck biopsy may have in fact slowed the diagnostic process.

Antiretroviral therapy was continued, but modified on the ICU. Notably the patient had been using antiretroviral therapy (HAART) for two years prior to her presentation to our service. She had had a persistently undetectable plasma HIV load (<50 copies/mL). Her nucleoside reverse transcriptase inhibitors (abacavir and lamivudine) were stopped because of their association with lactic acidosis and hepatic steatosis. Although it would be surprising for this to occur after such a prolonged time on these drugs, it was important to minimise any possible mitochondrial toxicity that might have resulted from these agents; and which in turn could have contributed to acidosis. Monotherapy with ritonavir-boosted protease inhibitors is a useful treatment option in patients who are intolerant or resistant to other agents [8]. The approach is particularly successful in patients who have already suppressed their plasma HIV load, such as in this case.

Her condition progressed rapidly to one of multi-organ failure requiring a high level of support on the ICU. Supportive strategies included mechanical ventilation for acute respiratory distress syndrome; circulatory support with inotropes/vasopressors and corticosteroids; and high volume haemofiltration for renal failure and severe metabolic acidosis. Corticosteroids were used empirically to treat probable underlying relative adrenal insufficiency. This occurs in up to 25% of critically ill patients with sepsis. Surviving Sepsis guidelines [9] published in 2004 do not suggest mandatory testing (by adrenocorticotrophic hormone: ACTH stimulation test) unless there is strong suspicion of undiagnosed primary adrenal insufficiency. The 10 mg/h infusion used in our patient was in line with the recommended 24 hour total dose of 200-300 mg. High volume haemofiltration was undertaken to target early shock reversal and removal of IL-6, increased expression of which is a hallmark feature of MCD. In animal studies, such a strategy is associated with improved haemodynamics and gas exchange, reduced immuno-paresis and increased survival [10]. The length of hospital stay post ICU discharge for our patient largely reflects the need for weaning from respiratory support and rehabilitation. Patients such as ours often experience profound muscle weakness (ICU-acquired weakness) after mechanical ventilation on intensive care, and require intensive physiotherapy, as well as nutritional, psychological and social support.

## Conclusion

Our patient's successful outcome can be attributed to relatively simple treatment for MCD: that is rituximab; and extensive multi-organ care support on the ICU. Rituximab is a newly-established therapy for MCD [1]. Its efficacy has thus far been chiefly demonstrated in limited studies of patients outside of the intensive care setting. Recent literature suggests ICU admission and support can be beneficial for haemato-oncological patients to facilitate specific chemotherapy [6]. Consistent with this notion, our report illustrates the benefit of ICU support during rituximab treatment for HIV associated MCD.

## Abbreviations

ACTH: adrenocorticotrophic hormone; AIHA: autoimmune haemolytic anaemia; APACHE II: acute physiology and chronic health evaluation II; APTT: activated partial thromboplastin time; ARDS: acute respiratory distress syndrome; CNS: central nervous system; DIC: disseminated intravascular coagulation; FiO_2_: fraction of inspired oxygen; HAART: highly active antiretroviral therapy; HHV-8: human herpes virus-8; HIV: human immunodeficiency virus; ICU: intensive care unit; MAP: mean arterial pressure; MCD: Multicentric Castleman's Disease; PT: prothrombin time; SAPS I: simplified acute physiology score I.

## Consent

Written informed consent was obtained from our patient for publication of this case report. A copy of the written consent is available for review by the Editor-in-Chief of this journal.

## Competing interests

The authors declare that they have no competing interests.

## Authors' contributions

All authors were involved in managing this case. RHJ wrote the manuscript with TD, with input from MCL, KC, SS and BA. JRC selected and provided radiology images. PGI examined histological specimens and provided images. All authors have read and approved the final manuscript.
